# Aminolevulinic Acid-Based Tumor Detection and Therapy: Molecular Mechanisms and Strategies for Enhancement

**DOI:** 10.3390/ijms161025865

**Published:** 2015-10-28

**Authors:** Xue Yang, Pratheeba Palasuberniam, Daniel Kraus, Bin Chen

**Affiliations:** Department of Pharmaceutical Sciences, Philadelphia College of Pharmacy, University of the Sciences, Philadelphia, PA 19104, USA; E-Mails: xyang1349@mail.usciences.edu (X.Y.); ppalasuberniam@mail.usciences.edu (P.P.); dkraus@mail.usciences.edu (D.K.)

**Keywords:** aminolevulinic acid (ALA), protoporphyrin IX (PpIX), photodynamic therapy (PDT), tumor detection, fluorescence, heme biosynthesis

## Abstract

Aminolevulinic acid (ALA) is the first metabolite in the heme biosynthesis pathway in humans. In addition to the end product heme, this pathway also produces other porphyrin metabolites. Protoporphyrin (PpIX) is one heme precursor porphyrin with good fluorescence and photosensitizing activity. Because tumors and other proliferating cells tend to exhibit a higher level of PpIX than normal cells after ALA incubation, ALA has been used as a prodrug to enable PpIX fluorescence detection and photodynamic therapy (PDT) of lesion tissues. Extensive studies have been carried out in the past twenty years to explore why some tumors exhibit elevated ALA-mediated PpIX and how to enhance PpIX levels to achieve better tumor detection and treatment. Here we would like to summarize previous research in order to stimulate future studies on these important topics. In this review, we focus on summarizing tumor-associated alterations in heme biosynthesis enzymes, mitochondrial functions and porphyrin transporters that contribute to ALA-PpIX increase in tumors. Mechanism-based therapeutic strategies for enhancing ALA-based modalities including iron chelators, differentiation agents and PpIX transporter inhibitors are also discussed.

## 1. Introduction

Considered as pigments of life, porphyrins attract tremendous human curiosity about their chemistry, biosynthesis, role in homeostasis and pathogenesis, and potential as therapeutic agents. As a class of tetrapyrroles with highly conjugated heterocyclic structure, porphyrins typically have intense absorption of light in the visible range, giving the characteristic red color in animals (due to heme) and green color in plants (due to chlorophyll). It was nearly 150 years ago that hematoporphyrin (HP), a crude porphyrin extract from blood, was first shown to have fluorescence property and about 100 years ago that the fluorescence of HP was found useful for detecting tumors [1]. The photosensitizing property of porphyrins, the ability to convert absorbed light energy into the production of cytotoxic reactive species in the presence of oxygen, was first recognized in the 1900s using HP and extensively studied since the 1970s using partially purified HP preparations [2]. These included hematoporphyrin derivatives (HPD) and Photofrin, which ultimately led to the world-wide approval of Photofrin-mediated photodynamic therapy (PDT) [1].

Because all porphyrins are biosynthesized from aminolevulinic acid (ALA), an early precursor in the heme biosynthetic pathway that is found in nearly all mammalian cells, ALA can be used to boost the production of endogenous porphyrins for many diagnostic and therapeutic uses. Following the pioneer work by Malik [3], Kennedy and Pottier [4] and Moan and Peng [5] who showed enhanced ALA-mediated protoporphyrin IX (PpIX) accumulation in tumor cells and effective cell destruction after light illumination, ALA was rapidly established as a promising PDT agent. With proven effectiveness in eliminating unwanted cells, good selectivity and excellent cosmetic effect, ALA-PDT received world-wide approval in the late 1990s and has become a mainstream treatment in dermatology [6]. Its applications in managing other types of cancers and non-cancer diseases are being actively explored as well [7,8]. Not only is it a remarkable PDT agent, ALA is also a useful imaging probe. With a broad red fluorescence emission extending close to the near-infrared region, ALA-mediated PpIX fluorescence is being used to guide the resection of brain and bladder tumors with encouraging clinical outcomes [9,10].

The key to the successful use of ALA as a PDT and imaging agent lies in the preferential accumulation of PpIX in target cells following ALA administration. Extensive research has been performed to determine the molecular mechanism involved in enhanced ALA-PpIX in tumor cells. In this special issue on Advances in PDT, we would like to summarize research on this topic over the past two decades. We begin with an overview of the biosynthesis and transport of PpIX, and then summarize current understanding on the mechanism involved in preferential ALA-mediated PpIX synthesis and accumulation in tumors. Next, we discuss mechanism-based therapeutic strategies for enhancing ALA-based tumor detection and PDT. Finally, we end with perspectives on areas for future studies. For readers who are interested in the clinical applications of ALA and its derivatives, there are excellent recent reviews [7,11,12,13] including one on ALA-PpIX fluorescence-guided glioblastoma resection in this issue [13]. We focus this review on the molecular mechanism underlying elevated ALA-PpIX in tumors and mechanism-based therapeutic approaches for enhancing ALA-based modalities with the goal of encouraging further research in these important areas.

## 2. Biosynthesis and Transport of PpIX

**Figure 1:**
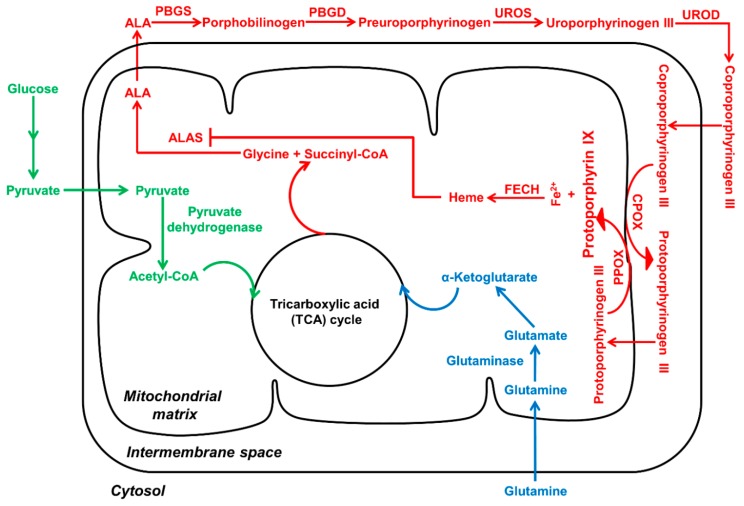
Heme biosynthesis pathway (in red) connects with glucose (in green) and glutamine (in blue) metabolic pathways. Porphyrin synthesis converges with energy metabolism through TCA (tricarboxylic acid) cycle. Enhanced glycolysis and glutaminolysis in tumor cells may activate heme biosynthetic pathway to ensure energy production and avoid the accumulation of TCA metabolites.

PpIX is a precursor to heme, the final product of the heme biosynthesis pathway (Figure 1). This pathway includes both mitochondrial and cytosolic processes catalyzed by a total of eight enzymes [14]. Seven enzymes are involved in PpIX synthesis and the last one converts PpIX to heme. The first enzymatic step in the heme biosynthesis pathway is the generation of ALA from glycine and succinyl coenzyme A by ALA synthase (ALAS) in the mitochondrion. ALA then migrates to the cytosol where two molecules of ALA are condensed to form porphobilinogen (PBG), the first monopyrrole in the pathway, in a reaction catalyzed by ALA dehydratase (ALAD), also known as porphobilinogen synthase (PBGS). Four molecules of PBG are connected to form hydroxymethylbilane (HMB), the first tetrapyrrole in the pathway, by porphobilinogen deaminase (PBGD), also known as hydroxymethylbilane synthase (HMBS). Linear tetrapyrrole HMB is closed to form cyclic uroporphyrinogen III by uroporphyrinogen III synthase (UROS). Decarboxylation of uroporphyrinogen III by uroporphyrinogen III decarboxylase (UROD) leads to coproporphyrinogen III. Coproporphyrinogen III is then transported back into mitochondria to undergo oxidative decarboxylation by coproporphyrinogen III oxidase (CPOX) to generate protoporphyrinogen III, which is further oxidized by protoporphyrinogen III oxidase (PPOX) to produce PpIX with aromatic chemical structure. PpIX is subsequently chelated with ferrous iron to form heme in mitochondria catalyzed by ferrochelatase (FECH) [14]. Interestingly, heme has essentially no fluorescence and photosensitizing activity, whereas PpIX possesses fluorescence and photosensitizing ability.

Because free porphyrin metabolites such as PpIX and heme are prone to cause oxidative stress and cell damage, cells maintain a low level of free porphyrins through mechanisms including well-coordinated heme biosynthesis and degradation, rapid utilization of free heme for hemoprotein synthesis, and extracellular transport of excess free porphyrins [15]. Heme biosynthesis is negatively regulated by the intracellular level of heme, which inhibits heme biosynthesis by imposing negative feedback inhibition on the rate-limiting enzyme ALAS and stimulates heme degradation by promoting the expression of heme degradation enzyme heme oxygenase [14]. Free heme is often rapidly committed to the formation of many heme-containing proteins such as hemoglobin and cytochrome P450 enzymes. Excess PpIX, heme and other porphyrins are efficiently transported out of cells through various heme transporters [15]. Although being lipophilic in general, PpIX and other porphyrins are negatively-charged molecules that require membrane transporters to facilitate movement across cell membranes. Among all cell membrane transporters that have been identified for the transport of heme and other porphyrins, the ATP-binding cassette sub-family G (ABCG) 2 protein, also known as breast cancer resistance protein (BCRP), has the most well-defined role in PpIX extracellular transport [16].

## 3. Mechanisms Involved in Enhanced PpIX Production and Accumulation

Elevated PpIX fluorescence is commonly observed in a variety of tumor cells and tissues following ALA administration compared with normal counterparts [7], which provides the basis for using ALA as a prodrug for fluorescence detection and photodynamic targeting of tumors. Thus, why tumor cells and tissues exhibit enhanced PpIX production and accumulation becomes a fundamental question associated with the application of ALA-based modalities in oncology. Although this remains an open question, extensive studies have suggested that increased PpIX fluorescence in tumor cells is likely a result of multiple tumor-associated cellular alterations including alterations in heme biosynthetic enzymes, mitochondrial functions and porphyrin transporters.

### 3.1. Alterations in Heme Biosynthetic Enzymes

Because PpIX is a metabolite produced in the heme biosynthetic pathway, it is reasonable to assume that the enhanced level of PpIX in tumor cells after ALA incubation is due to changes in the expression or activity of heme biosynthesis enzymes in tumor cells. Comparing the expression or activity of heme biosynthesis enzymes between tumor and normal cells or tissues does reveal significant differences in some cases. However, conflicting results, often dependent on tumor type, are commonly seen in the literature.

*ALAS* gene expression was compared between micro-dissected tumor tissues from colorectal cancer patients and corresponding normal tissues by RT-PCR [17]. *ALAS* expression in tumor tissues was significantly lower than in normal tissues. However, both *ALAS* gene expression and protein level were found increased in HCC4017 non-small-cell lung cancer (NSCLC) cells compared with normal cells, and increased ALAS protein level was shown in a panel of human lung cancer xenograft tumor samples [18]. It should be pointed out that changes in ALAS expression and activity are not expected to affect ALA-mediated PpIX production because exogenous ALA bypasses this enzymatic step.

PBGD has been suggested to play an important role in elevated ALA-PpIX production in tumor cells based on the findings that increased *PBGD* expression or activity is associated with cell transformation [19,20], and the upregulation of PBGD enzymatic activity has been found in some cancer cells [21,22,23] and after ALA stimulation [24,25]. There are case studies suggesting the involvement of high *PBGD* gene expression or enzyme activity in the oncogenesis of cervical [26], prostate [27] and breast [28] cancers, and meningioma but not glioma [29]. Significantly higher than normal PBGD activity has been found in human bladder [22] and colon [23] cancer cells as well as in tissue samples from Barrett’s esophagus and esophageal cancer patients [21,30], although there is no significant difference in *PBGD* expression between human colorectal cancer and normal tissues [17]. Because increased PBGD activity and decreased FECH activity were detected in some esophageal tumor cells/tissues, the ratio of PBGD to FECH activity was proposed as an index to predict enhanced PpIX accumulation and cell sensitivity to ALA-PDT [21]. However, subsequent studies failed to demonstrate the predictive value of this index [22,23,30]. In addition, overexpression of *PBGD* does not result in increased PpIX production, suggesting a complex interplay between PBGD and other heme biosynthesis enzymes [25,31].

There is evidence showing the involvement of increased *UROD* gene expression or enzyme activity in tumor initiation and progression. *UROD* was highly expressed at an early stage, but not at a late stage, in Friend virus-induced erythroleukemia in mice [32]. Enhanced UROD activity together with increased porphyrin biosynthesis were detected in human breast tumor tissues compared with normal tissues [28]. *UROD* expression was significantly elevated in tumor biopsies from head and neck cancer patients [33]. In addition, patients with higher *UROD* expression were more likely to have shorter disease-free survival, suggesting the involvement of UROD in cancer progression. However, in human clear-cell renal carcinomas, there was no correlation between the UROD activity and the concentration of total porphyrins or the degree of malignancy [34].

As an enzyme responsible for converting PpIX to heme, *FECH* gene expression or enzyme activity has often been found reduced in a variety of tumor cells/tissues including liver [35], bladder [22], colorectal [17,23], esophageal, gastric and rectal cancers [17] compared with normal counterparts. Cell lines with reduced FECH level or activity tend to have a higher ALA-PpIX level while cells with increased FECH level or activity are more likely to exhibit a lower ALA-PpIX fluorescence [17,36]. Silencing *FECH* gene expression significantly increased ALA-PpIX fluorescence in human colon [17], urothelial [37], glioma [38] and breast [39] cancer cells and sensitized cells to ALA-PDT whereas overexpression of *FECH* reduced cell sensitivity to ALA-PDT by decreasing PpIX production [36]. Although reduced FECH expression/activity is often associated with enhanced ALA-PpIX in tumors, there are reports showing good ALA-PpIX production in tumors without reduced FECH activity [30,40,41], suggesting the involvement of other contributing factors.

### 3.2. Alterations in Mitochondrial Functions

The observation that PpIX is often accumulated in tumor cell mitochondria leads to a speculation that enhanced ALA-PpIX is related to mitochondrial alterations in tumor cells. Comparing mitochondrial content (as indicated by the fluorescence intensity of MitoTracker dye) and the activity of cytochrome c oxidase (a mitochondrial enzyme involved in oxidative phosphorylation) with ALA-PpIX level in different tumor cell lines indicates a correlation between ALA-PpIX level and mitochondrial content, but not with cytochrome c oxidase [41]. However, later studies involving more cell lines demonstrate that such a simple correlation between ALA-PpIX and mitochondrial content or the activity of certain mitochondrial enzymes often cannot be established [23,42,43].

Recent studies suggest that alterations in energy metabolism, particularly enhanced glycolysis, are involved in enhanced ALA-PpIX in tumor cells (Figure 1). Similar to PpIX/heme biosynthesis, glucose metabolism also has both cytosolic and mitochondrial processes. Succinyl-CoA, a metabolite produced in the glucose tricarboxylic acid (TCA) cycle in mitochondria, is one of two starting substrates for PpIX/heme synthesis. The crosstalk between glucose metabolism and porphyrin biosynthesis has been demonstrated by a recent finding that inactivation of TCA cycle activated heme biosynthesis in order to avoid the accumulation of TCA cycle metabolites and enable mitochondrial NADH production [44]. Because cancer cells often switch from the TCA cycle to aerobic glycolysis and glutamine for energy production [45], such metabolic reprogramming may lead to the accumulation of TCA cycle metabolites, which activates heme biosynthesis pathway to remove TCA metabolites. This may result in enhanced PpIX accumulation due to the saturation of FECH. Although more studies are needed to test this hypothesis, the connection between cancer cell metabolic reprogramming and ALA-PpIX accumulation is supported by another recent study where human glioma cells with mutated TCA cycle enzyme isocitrate dehydrogenase 1 (IDH1) exhibited enhanced ALA-PpIX as compared to cells with wide type IDH1 [46].

### 3.3. Alterations in Porphyrin Transporters

ALA-mediated PpIX synthesis depends on active ALA uptake and effective transport of different porphyrin metabolites between the cytosol and mitochondria by porphyrin transporters [15]. Porphyrin importers ensure porphyrin substrates are transported to the right intracellular site for porphyrin synthesis, while porphyrin exporters pump excess heme/porphyrins out of organelles or cells to maintain homeostasis. Theoretically, enhanced ALA-PpIX in tumor cells can be caused by factors including elevated ALA uptake, enhanced porphyrin importer activity and decreased PpIX exporter activity. Although enhanced ALA uptake has been shown in tumor cells with elevated PpIX [36], more studies demonstrate that the difference in ALA uptake between higher and lower PpIX producing cell lines is not significant [23,41,47] and the rate of ALA uptake is far greater than PpIX synthesis [48], indicating that ALA uptake does not appear to be a determining factor for enhanced ALA-PpIX in tumor cells.

One porphyrin transporter involved in PpIX synthesis is the ATP-binding cassette sub-family B member 6 (ABCB6) [49]. Originally identified as a transporter on the outer mitochondrial membrane, ABCB6 binds to various porphyrins including coproporphyrinogen III, PpIX and heme. Because it has the highest affinity to coproporphyrinogen III, ABCB6 is thought to be primarily involved in transporting coproporphyrinogen III into mitochondria for PpIX/heme synthesis [49]. Human glioma tumors show higher *ABCB6* expression than normal brain tissues [50]. The notion that increased ABCB6 function plays an important role in enhanced ALA-PpIX levels in tumor cells is supported by the findings that human glioma tissues with higher ALA-PpIX fluorescence exhibit higher *ABCB6* expression than glioma tissues with lower PpIX fluorescence, and *ABCB6* overexpression significantly increases ALA-PpIX fluorescence in glioma cell lines [50]. However, ABCB6 has also been shown to localize to the cell membrane [51] and endoplasmic reticulum [52], and transport coproporphyrinogen III out of cells [53], suggesting that enhanced ABCB6 function on cell membrane could potentially reduce PpIX/heme level by decreasing the intracellular concentration of coproporphyrinogen III. The net effect of ABCB6 on ALA-PpIX level is likely dependent on the relative ABCB6 activity in the mitochondrial *versus* cell membrane.

As a drug efflux transporter responsible for cancer cell resistance to many anticancer agents, the ABCG2 transporter probably plays the most important role in the extracellular transport of PpIX. Knockdown of *ABCG2* causes PpIX accumulation, particularly in mitochondria, and results in mitochondrial damage, indicating that PpIX is an endogenous substrate of ABCG2 [54,55]. The ABCG2 transporter is localized to both mitochondrial and cell membrane, which enables the extracellular transport of PpIX synthesized in mitochondria [56]. Increased ABCG2 activity has been shown to decrease intracellular PpIX level after ALA stimulation [16] and cell lines with high ABCG2 expressions or activities often exhibit reduced ALA-PpIX fluorescence [57]. In a recent study, we found that triple negative breast cancer cell lines have significantly reduced ALA-PpIX levels as compared with estrogen receptor (ER) positive and human epidermal growth factor receptor 2 (HER2) positive breast cancer cell lines because of elevated ABCG2 activity [58].

## 4. Therapeutic Strategies for Enhancing ALA-Based Tumor Detection and Therapy

**Figure 2:**
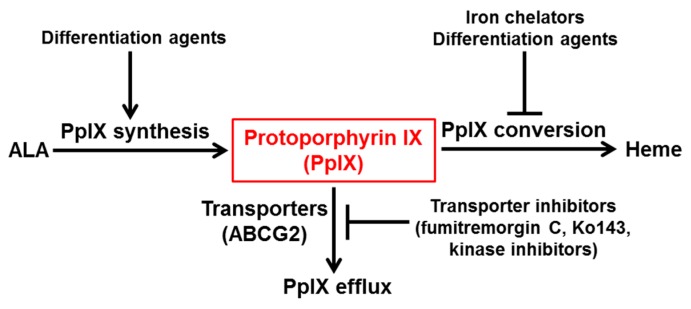
Current therapeutic strategies for enhancing ALA-based tumor detection and therapy. These strategies include enhancing PpIX synthesis, reducing PpIX conversion and inhibiting PpIX efflux.

Although ALA-based modalities have been used in the clinic for detecting and targeting tumor tissues, its applications are limited by inadequate and heterogeneous PpIX production in tumor cells [59,60]. Thus, various therapeutic approaches have been proposed and evaluated to overcome these limitations. All enhancement approaches can be categorized into three therapeutic strategies, which are: enhancing PpIX synthesis, reducing PpIX conversion, and inhibiting PpIX efflux (Figure 2). Enhancing PpIX synthesis can be achieved by increasing the activity of enzymes involved in PpIX synthesis and the transport of porphyrin intermediates necessary for PpIX synthesis. Reducing PpIX conversion aims at inhibiting the bioconversion from PpIX to heme by removing the substrate ferrous ion required for the reaction and/or inhibiting the enzyme FECH that catalyzes the reaction. Inhibiting PpIX efflux is to prevent PpIX extracellular transport with inhibitors of PpIX transporters, the ABCG2 transporter in particular.

It is desirable for a therapeutic agent to enhance ALA-PpIX level through multiple mechanisms. For example, differentiation agent vitamin D is able to enhance PpIX synthesis by up-regulating CPOX and reduce PpIX conversion by down-regulating FECH [61]. It is important to point out that the therapeutic potential of some enhancement approaches has been validated by genetic approaches. For instance, transfection of mutated *ALAS2* gene variants with increased enzymatic activity than the wild-type enzyme [62], as well as the *CPOX*-overexpressing vector [63], significantly increase PpIX production. Silencing *FECH* [17,38,39] and *ABCG2* [55] gene expression has been shown to increase PpIX accumulation. Here we summarize the therapeutic agents that have been evaluated for enhancing ALA-based tumor detection and therapy with promising results.

### 4.1. Iron Chelators

Because ferrous iron is a substrate necessary for converting PpIX to heme by FECH, removal of ferrous iron by iron chelators prevents this conversion, resulting in PpIX accumulation. The non-specific metal ion chelator ethylenediamine tetraacetic acid (EDTA) was first used to enhance ALA-PpIX accumulation in leukemia cells [64]. A more potent and selective iron chelator deferoxamine (DFO) [65] was later found to increase ALA-PpIX accumulation and sensitivity to ALA-PDT in a variety of tumor cells and tissues [66,67,68,69,70,71]. However, in a small scale clinical study, DFO showed no enhancement of ALA-PpIX and PDT in patients with superficial basal cell carcinomas or Bowen’s disease, and was only able to increase ALA-PpIX in normal skin at a low, but not high, ALA dose [72].

CP94 (1,2-diethyl-3-hydroxypyridin-4-one hydrochloride) is another iron chelator that is superior to DFO in enhancing ALA-PpIX accumulation likely due to its lower molecular weight and higher lipophilicity, resulting in better tissue penetration [73]. CP94 is able to enhance ALA-PpIX accumulation in rat bladder urothelium [74] and colonic mucosa [75] *in vivo*. It increases PpIX levels in human skin [73,76], bladder [77] and glioma [76,78] cancer cells treated with ALA or its derivatives. The safety and effectiveness of CP94 in enhancing PDT with methyl-aminolevulinate (MAL) for basal cell carcinoma has also been demonstrated in a pilot clinical study [79], indicating the feasibility of adding iron chelators into clinical PDT protocols.

### 4.2. Differentiation Agents

Induction of keratinocyte differentiation by increasing calcium concentration in medium enhances ALA-PpIX production and cytotoxicity to ALA-PDT through increased ALA uptake, increased PpIX synthesis by elevating CPOX expression, and decreased PpIX efflux [80]. Following this early study, differentiation agents including methotrexate (MTX) and vitamin D have been extensively studied for enhancing ALA-PpIX production and ALA-PDT outcomes [81]. Vitamin D and MTX increase PpIX fluorescence and cytotoxicity to ALA-PDT in LNCaP prostate cancer cells by up-regulating CPOX and differentiation marker E-cadherin [63,82]. It appears that differentiation-induced CPOX up-regulation is a major contributing factor for enhancing ALA-PpIX production because *CPOX* over-expression alone is enough to increase PpIX fluorescence [63].

Both the active form of vitamin D (calcitriol) [61] and dietary vitamin D (cholecalciferol) [83] can enhance ALA-PpIX production and PDT-induced cell death in A431 tumor cells and tissues. Calcitriol has also been shown to enhance ALA-PpIX and PDT in the MDA-MB-231 breast tumor model [84] and is effective for detecting mouse skin tumors based on enhanced ALA-PpIX fluorescence [85]. Calcitriol treatment increases E-cadherin and Ki67 staining, up-regulates CPOX and down-regulates FECH, leading to ALA-PpIX accumulation [61]. Furthermore, it increases TNF_α_ level, which may enhance extrinsic apoptosis induced by ALA-PDT [61]. The mechanism involved in calcitriol-induced CPOX up-regulation has been found to be related to the activation of transcription factor CCAAT enhancer binding proteins (CEBPs), which leads to the activation of its downstream *CPOX* gene transcription [86].

### 4.3. ABCG2 Transporter Inhibitors

The identification of PpIX as an endogenous substrate of ABCG2 transporter leads to the use of ABCG2 transport inhibitors to enhance ALA-PpIX fluorescence and PDT effects [16]. Increased ALA-PpIX accumulation in various tumor cell lines by inhibiting PpIX efflux has been demonstrated with ABCG2 inhibitors fumitremorgin C [16,87] and its less toxic and more selective analog Ko143 [57,58,88]. Because the activity of ABCG2 transporter depends on albumin, the effect of ABCG2 inhibitor on PpIX increase is particularly pronounced when cells are cultured in serum-containing medium and barely noticeable when serum-free medium is used. [87,89]. It is therapeutically important that some approved tyrosine kinase inhibitors including imatinib mesylate (Gleevec) [90] and gefitinib [91] are potent ABCG2 transporter inhibitors and effective in sensitizing tumor cells to ALA-PDT by increasing PpIX level. Moreover, the fact that ABCG2 inhibitor Ko143 is able to reduce PpIX fluorescence heterogeneity in tumor cells suggests that ABCG2 is involved in the intra-tumor heterogeneity of ALA-PpIX [58]. Finally, the effect of ABCG2 inhibitors on ALA-PpIX increase was observed only in cells with ABCG2 expression or activity, but not in cells lacking ABCG2 expression or activity, indicating the selectivity of this enhancement approach [57,58,90].

## 5. Conclusions and Future Perspectives

The past two decades have witnessed extensive research aiming at unraveling the mystery of enhanced ALA-PpIX in tumors and seeking therapeutic approaches to boost PpIX levels in tumors with insufficient PpIX accumulation. As a result of this intensive study, considerable knowledge has been obtained about various tumor-related pathological alterations that contribute to an increased level of PpIX in tumors following ALA stimulation. This knowledge has provided the foundation for the clinical application of ALA for detecting and targeting tumors, particularly in the skin, brain and bladder. It has also stimulated the exploration of mechanism-based therapeutic approaches to enhance ALA-based modalities, which has led to many encouraging preclinical and clinical results.

However, the molecular mechanism underlying enhanced ALA-PpIX in tumor cells remains elusive and those promising results are yet to be translated to clinical practice. To fulfill the potential of ALA-based modalities, more mechanistic basic research, well-designed clinical trials, and collaborative studies between bench scientists and clinicians are needed. In basic research, many cellular and functional alterations that contribute to an enhanced ALA-PpIX level in tumors have been identified. But we lack a comprehensive understanding of the underlying connection between these different pathological alterations and the relationship between these alterations and cell phenotypic changes (gaining survival advantage, metastatic potential, avoiding metabolic catastrophe *etc.*). More importantly, we do not know the cause of these pathological alterations in tumor cells. Is enhanced ALA-PpIX directly caused by oncogene activation in tumor cells? If so, what oncogenes are more likely to enhance tumor PpIX level and how? Obviously, answers to these fundamental questions will not only help us understand the biological meaning of increased ALA-PpIX level in tumors, but also have important implications in selecting appropriate patients for ALA-based modalities.

In clinical research, it is certainly encouraging to see promising results from pilot clinical studies where ALA-PpIX is used for diagnosing tumors other than glioblastoma, and iron chelators are able to improve PDT outcome. However, these clinical studies are limited by very small sample size and sometimes patients with mixed diseases. Well-designed large-scale clinical trials that will eventually convince the medical community and regulatory agencies to accept these new ALA-based therapeutic modalities are needed. Finally, in the field of oncology where personalized medicine is taking the lead, the importance, as well as the benefit, of collaborative research between bench scientists and clinicians cannot be overemphasized. ALA is certainly not good for all types of cancer nor for all patients with a certain type of cancer. Identifying the appropriate disease and patient subsets who will benefit the most from ALA-based modalities is probably the most important task facing researchers in this field in the next decade. Through more collaborative research between basic and clinical scientists, it is hoped that we will have more mechanistic understanding of ALA-based modalities and more patients will benefit from these modalities.
